# Climate change-driven geographical shifts in *Aspergillus* species and the implications for plant and human health

**DOI:** 10.1016/j.isci.2026.114911

**Published:** 2026-02-05

**Authors:** Christopher Uzzell, Jennifer Shelton, Norman van Rhijn

**Affiliations:** 1 Liverpool School of Tropical Medicine, Liverpool, UK; 2 UK Centre for Ecology & Hydrology, Wallingford, UK; 3 Manchester Fungal Infection Group, Division of Evolution, Infection, and Genomics, Faculty of Biology, Medicine and Health, University of Manchester, Manchester, UK; 4 Microbial Evolution Research Manchester, Division of Evolution, Infection, and Genomics, Faculty of Biology, Medicine and Health, University of Manchester, Manchester, UK

**Keywords:** Environmental health, Global change, Mycology

## Abstract

*Aspergillus* species cause severe infections and are widespread environmental saprotrophs. Climate change is expected to alter the ecological niches and spread of fungal pathogens. Here, we use a global metabarcoding dataset and Maximum Entropy (MaxEnt) modeling to predict the current and future environmental suitability of three pathogenic *Aspergilli*: *A. fumigatus sensu lato*, *A. flavus sensu lato*, and *A. niger sensu lato*. We show that the suitability of *A. fumigatus* is higher in temperate climates, while *A. flavus* and *A. niger* are more suitable in warmer regions. Future climate scenarios suggest a northward shift of habitat suitability for all three species, particularly under severe warming. We combine our MaxEnt model with spatial models of crop growing areas and human population, and show that geographical shift will occur on *Aspergillus* species along different climate scenarios. These predictions can guide experimental validation efforts and provide a base model for further refinement for other pathogenic fungi.

## Highlights

• MaxEnt models reveal poleward habitat shifts of *Aspergillus* spp. under climate change
• *A. fumigatus* prefers temperate, while *A. flavus* and *A. niger* thrive in warmer climates
• Climate-driven habitat shifts reduce overlap with maize and rice crop-growing areas
• Clinical prevalence of aspergillosis mirrors environmental suitability patterns

## Graphical abstract

**Figure:**
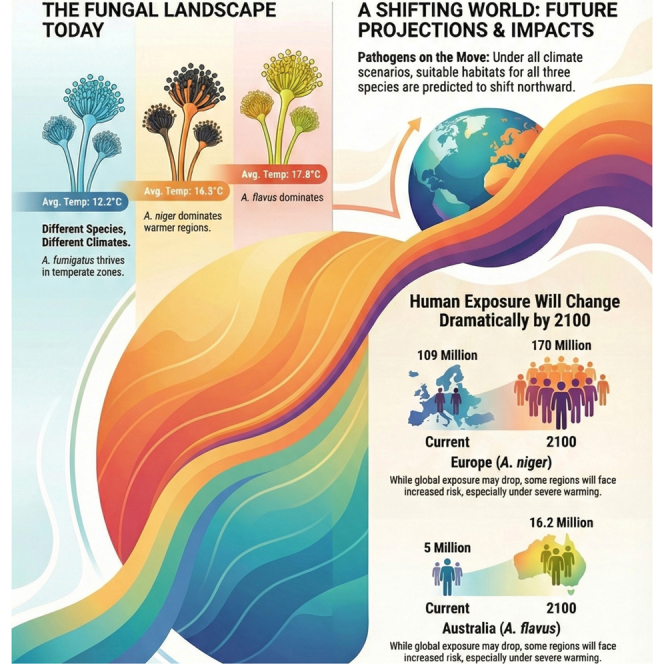

## Introduction

The filamentous fungal *Aspergillus* species are the prime example of a cross-kingdom pathogen. They are capable of infecting humans, other mammals, birds, honeybees, and corals, they spoil crops pre- and post-harvest, and they render crops unsafe for consumption by producing mycotoxins.^1^^,^^2^ They also play a crucial role in the environment as saprotrophs, recycling nutrients in decaying matter back into the soil.^3^ Furthermore, frontline drugs used to treat clinical and veterinary aspergillosis, namely azoles, are also found in agricultural pesticides used to protect crops against fungal disease.^4^^,^^5^^,^^6^ The structural similarity between clinical azoles and agricultural azoles has led to a rise in patients with azole-resistant infections after inhaling *Aspergillus* spores that have developed resistance following environmental exposure to azoles.^7^^,^^8^^,^^9^

Due to their life cycle, reproducing asexually and sexually in soil and sporulating to release 1000s of microscopic spores, *Aspergillus* spores are ubiquitous in the air.^10^^,^^11^ They are found indoors and outdoors, are detectable on a global scale, and it is estimated that we each inhale several hundred spores per day.^12^^,^^13^ The small size of these spores (2–3 μm) allows them to bypass mucociliary clearance and reach the lung alveoli, where they are subsequently cleared by the innate immune system.^14^ However, in individuals with a compromised immune system, or who have been exposed to a high number of spores, spores can establish and grow in a pre-existing cavity in the lung, resulting in chronic pulmonary aspergillosis (CPA).^15^^,^^16^ If the immune system fails to prevent spores from entering the bloodstream via the lungs, the infection results in a life-threatening disease called invasive aspergillosis (IA).^17^ It is estimated that 1.8 million people globally develop CPA, with 340,000 annual deaths, and 2.1 million people globally develop IA, with 1.8 million annual deaths.^18^

There are a number of *Aspergillus* species more commonly associated with aspergillosis infections in humans and animals: *A. fumigatus, A. flavus, A. niger*, *A. terreus,* and *A. nidulans*.^19^ In the Northern Hemisphere, the majority of aspergillosis infections are caused by *A. fumigatus*,^20^^,^^21^^,^^22^^,^^23^ which in part contributed to the World Health Organisation (WHO) adding *A. fumigatus* to its fungal priority pathogens list (FPPL).^24^ However, in other parts of the world, other *Aspergillus* species are often reported as the leading cause of aspergillosis.^25^^,^^26^^,^^27^ It is likely that environmental conditions, such as temperature, humidity, and rainfall, favor the proliferation of different *Aspergillus* species in different climates.^28^^,^^29^^,^^30^^,^^31^ It has been hypothesised that climate change will bring about an increase in human fungal infections in multiple ways, including: (i) by increasing the range of currently pathogenic species, and (ii) by increasing the thermotolerance of fungal species, allowing more to survive at mammalian body temperature.^32^^,^^33^^,^^34^^,^^35^^,^^36^ It follows that climate change may alter the distribution of currently pathogenic *Aspergillus* species, or enable other *Aspergillus* species to become pathogenic, leading to late or underdiagnosis of aspergillosis infections caused by unexpected species.

The same logic applies to *Aspergillus* species that cause crop losses, either through spoilage or mycotoxin contamination.^37^ We are currently facing the challenge of feeding a predicted population of 9.7 billion by 2050, yet we still lose 20% of crop yields pre-harvest and a further 10% post-harvest to pathogens.^38^^,^^39^ Black *Aspergillus* species, such as *A. niger*, and *Aspergillus* section *Flavi*, which includes *A. flavus*, are the most often reported plant-pathogenic *Aspergilli*.^40^ It is estimated that aflatoxin contamination could cost the corn industry in the United States alone between US$52.1 million and US$1.68 billion, with the upper estimate for if climate change causes more regular aflatoxin contamination in the Corn Belt as was experienced in 2012.^41^

Studies of other fungal pathogens have underscored the significance of environmental conditions in shaping host-pathogen dynamics. *Cryptococcus neoformans* is a significant fungal pathogen of humans that is adapted to grow in warmer environments. Some strains of this organism have acquired enhanced thermotolerance, which enhances their virulence.^42^^,^^43^ Likewise, *Fusarium* species that damage both plants and humans adaptively respond to climate variations with increases in toxin production and fungicide resistance under warmer temperatures.^44^^,^^45^ It is timely that we build a global picture of *Aspergillus* species distribution: to understand what it looks like now and predict what it might look like in the future, based on the known impacts of climate variables on spore proliferation. In this study, we use a literature review and the GlobalFungi database to ascertain the current distribution of three pathogenic *Aspergillus* species: *A. fumigatus, A. flavus* and *A. niger,* and MaxEnt modeling to predict how the distribution of these species might alter in future climate scenarios.

## Results

### A maximum entropy model shows the geographic expansion of *Aspergillus* species

It has been hypothesised that fungal pathogens will expand their geographical range due to climatic changes within the next 100 years. However, currently there is little data to support these statements as experimental validation would rely on long-term standardised global sampling efforts. Therefore, we approached this hypothesis using available metabarcoding sequencing data from GlobalFungi^46^ and Maximum Entropy modeling. We focused on three fungal pathogens within the *Aspergillus* genus, as these are causative agents of human infections, but also plant infections. From the GlobalFungi database, we obtained metabarcodes that both included ribosomal ITS1 and ITS2 data. These metabarcodes are only able to accurately define the three *Aspergillus* species up to their section level: *Aspergillus* Section *Fumigati*, *Aspergillus* Section *Nigri,* and *Aspergillus* Section *Flavi*.^47^^,^^48^ However, speciation within these sections relies on multiple genetic markers (calmodulin and beta-tubulin), which are not available within the GlobalFungi dataset. Therefore, in here we refer to these further as *Aspergillus fumigatus sensu lato*, *Aspergillus niger sensu lato,* and *Aspergillus flavus sensu lato*. We obtained data from 2599 samples, 5124 samples, and 4015 samples for *Aspergillus niger*, *Aspergillus flavus,* and *Aspergillus fumigatus*, respectively. After quality control, 1021, 871, and 319 datapoints were considered of high quality and contained all required metadata for *Aspergillus niger*, *Aspergillus flavus,* and *Aspergillus fumigatus*, respectively (Figure S1). We only included datapoints from natural soil samples (non-experimental). Several unique biomes were not represented in this data, as sampling data of these regions is sparse. Data from the Amazon region, the Sahara, and northern Russia and Alaska were not available, and therefore, we can not make any accurate predictions for these regions. Latitude and longitude data of occurrences were used in the MaxEnt model together with bioclimatic variables. We assessed the correlation between bioclimate variables to reduce the variables, autocorrelation, and overfitting in the model (Figure S2). ROC curves were obtained to quantify the model's predictive ability relative to a random prediction (Figure S3). For *A. flavus,* the AUC of the ROC curve was 0.804, for *A. fumigatus,* 0.874, and for *A. niger,* 0.776, showing that the MaxEnt model predicts suitable habitat better than a random model. Furthermore, the jackknife test on the regularized training data showed that the annual mean temperature was considered the most important variable when taken in isolation for all three fungi (Figure S3). In a multivariate model that included the 7 bioclim variables, omission of the annual mean temperature reduced the fit of the model of *A. flavus* and *A. fumigatus* habitat, while the omission of precipitation of the coldest month most reduced the fit of the model for *A. niger* habitat.

In addition, we extracted the data from the SoilGrid database on pH, sand, clay, and silt particles, organic carbon stocks and density, total nitrogen, water retention, bulk density, and cation exchange capacity to give further granularity to the metabarcoding data.^49^ This revealed that different patterns for each species could be found in cation exchange capacity, where *A. flavus* was present in higher cmol(c)/kg soil compared to the other two species (Figure S4). In addition, *A. flavus* was found more in soil with lower carbon and nitrogen content, and in soil with a higher proportion of sand particles, which is in line with the published literature.^50^^,^^51^ *A. fumigatus* was more commonly found in soils containing higher levels of nitrogen and carbon stocks, a higher proportion of clay particles, as well as lower pH soils. These findings are also in line with previously published reports.^52^^,^^53^^,^^54^

**Figure 1:**
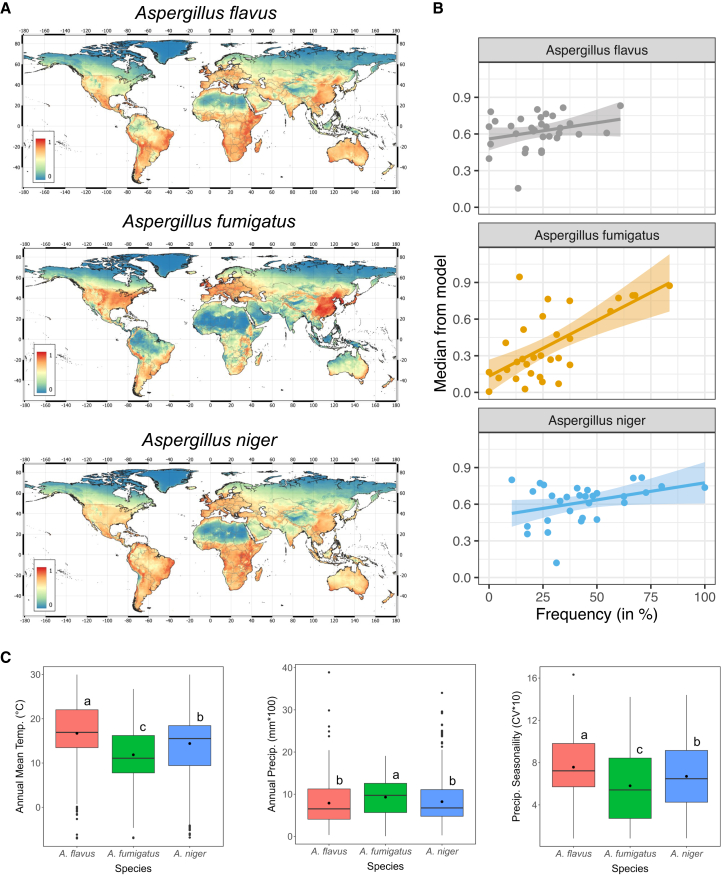
MaxENT model accurately described *Aspergillus* global distributions (A) Habitat suitability for three *Aspergillus* species from the MaxENT model. Least suitable is 0 and most suitable is 1. (B) Correlation plots of the frequency of each *Aspergillus* species found in the literature compared to the median suitability for that particular country in the MaxENT model. Shaded region represents the 95% confidence interval. (C) Boxplots show environmental differences between *A. flavus, A. fumigatus, and A. niger* among three environmental variables used for SDM. Species marked with the same letter are not significantly different at *p* < 0.05 with regard to each environmental variable. Boxplot shows the median and interquartile ranges. Whiskers represent the lower and upper quartile range. a shows significance (*p* < 0.05) versus the two other groups, b significance (*p* < 0.05) versus *Aspergillus fumigatus,* and c versus *Aspergillus flavus* as determined by one-way ANOVA.

The MaxEnt model resulted in a world map with the suitability profile for each of these fungi (Figure 1A). Not completely unsurprisingly, *A. fumigatus* was most suitable in the northern hemisphere in temperate climates, and *A. flavus* was more suitable for tropical regions. As relative abundance within each sample was not taken into consideration, we divided the habitat into two categories: suitable and unsuitable. We used the maximum test sensitivity plus specificity (MTSPS) as a threshold for suitable and non-suitable, which is generally used to classify suitability in MaxENT modeling.^55^^,^^56^ A cut-off above 0.64, 0.61, and 0.68 was considered suitable habitat for *A. flavus*, *A. niger,* and *A. fumigatus*, respectively. To validate our model, we collected published data on culture-based experimentation where culturing from soil was performed, which allowed the identification of the three *Aspergillus* species. We chose soil culture experiments only, as *Aspergilli* species are ubiquitously found in the air. This resulted in 29 studies from different countries that identified all three *Aspergillus* species from soils. A positive correlation could be found between suitability from the MaxEnt model and frequency of each species from culture-based experimentation found in each study (r(91) = 0.44, *p* = 0.114 x 10^−5^) (Figure 1B).

Within our species distribution model environmental variables that significantly differed between the *Aspergillus* species included the annual mean temperature, annual precipitation and precipitation seasonality (Figure 1C). *A. flavus* and *A. niger* showed presence at a significantly higher annual mean temperature compared to A. fumigatus, at 17.8 and 16.5 and 12.3 average Celsius, respectively (one-way ANOVA with multiple comparison, *p* < 0.05) (Figure S5). Significantly higher precipitation was associated with the presence of *A. fumigatus* compared to *A. flavus* and *A. niger*, while higher seasonality of precipitation was associated with *A. flavus* and *A. niger* (Figure S5).

### Expanding geographic ranges can impact the spectrum of aspergillosis disease in plants and humans

Next, we wanted to model the environmental suitability changing over time due to climatic changes. We used the shared socioeconcomic pathways (SSP) models SSP126, SSP245, and SSP585 within three time horizons (2041–2060, 2061–2080, 2081–2100) to assess the changes in suitability for the three *Aspergillus* species. The SSP126 model is the low emissions scenario, where the focus lies on a future with sustainability-focused development, where CO_2_ emissions decline after 2025 and limit global warming to below 2°C. The SSP245 model is the intermediate emissions scenario where CO_2_ emissions peak around 2040 and then decline slowly, with global warming reaching 2.5°C–3°C by 2100. The SSP585 is the high emissions model where fossil-fuel driven development is central, and CO_2_ emissions keep rising. This scenario would see warming of 4 °C or more by 2100. Of these SSP models and time horizons, all bioclimatic variables contributing to the model were included. Using the MaxEnt model, we generated a habitat suitable/non-suitable map for these climate change models until 2100 (Figure S6).

**Figure 2:**
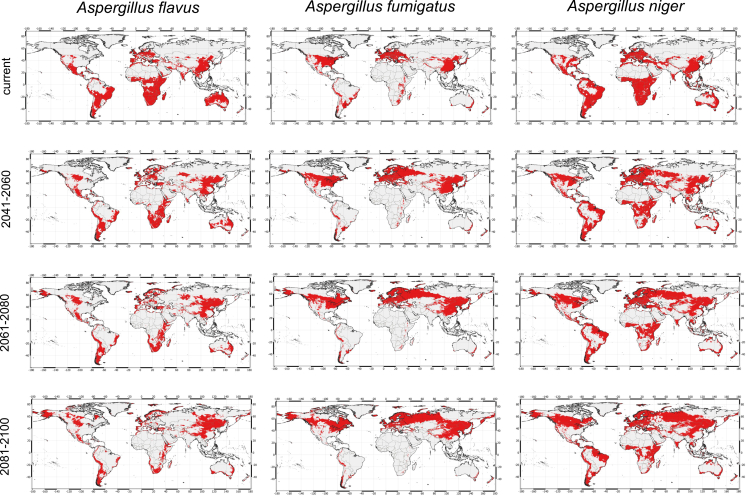
Climate change will shift distributions of *Aspergillus* species The SSP245 model is shown here as a representative across three different time horizons (2041–2060, 2051-200, and 2081–2100). Red is considered a suitable habitat according to the cut-off from the MTSPS analysis.

For *A. flavus,* the current suitable habitat contains much of the middle of South Africa, Brazil, and part of Mexico, large parts of South America, India, Pakistan, China, and South-East Asia, as well as Oceania. Under the low climate change model (SSP126), little will change for the habitat suitability of *A. flavus* until 2100, and most regions will remain suitable, while only small pockets of land will become more suitable (Figure S6). Under the moderate model (SSP245) (Figure 2), habitat suitability in Australia will largely disappear by 2100 while new suitable habitats are seen in north China and across Russia and part of northern America. Under the severe model (SSP585), by 2100, many of the suitable habitats will disappear, mainly on the African continent and across Brazil (Figure S6). Large parts of Australia will become unsuitable. However, larger parts of north China and Russia will become suitable as well as other parts of the northern hemisphere, such as Scandinavia and Alaska. This is supported by looking at suitability across latitude, where 40 to 80° latitude will become more suitable while 20 to −20 latitude will become less suitable (Figure S7).

*Aspergillus fumigatus's* suitable habitat is currently mostly in the northern hemisphere in Europe, the United States, and parts of China. However, in the southern hemisphere, parts of Brazil and Africa are also considered suitable, as is New Zealand, and some coastal regions in Australia. Under the low climate change model and moderate models (SSP126 and SSP245) only small parts of the northern hemisphere will become suitable for *A. fumigatus,* and little change will be in the southern hemisphere's suitable areas (Figure 2). However, under the severe model (SSP585), *A. fumigatus* suitable habitats will almost exclusively be in the northern hemisphere and pushed more toward the north pole (Figure S6). Still, New Zealand, coastal Australia, parts of Argentina, and Peru will be suitable as these remain more temperate climates. This is supported by suitability across latitudes as a strong decrease of suitability is observed from 40 to −40° latitude (Figure S7).

*Aspergillus niger* habitat is currently suitable across many regions of the world, including all continents and many countries in the northern and southern hemispheres. None of the climate models will have a drastic impact on the northern hemisphere suitability for *A. niger*. The suitability for the southern hemisphere, in particular Africa, will change only in the land-inward region under the severe climate model (SSP585), but is predicted to remain suitable along the coastal regions (Figure S6). Suitability along latitude supports this as only marginal decreases are seen from 0° to −40 latitude and some small increases from 50 to 80° latitude (Figure S7).

**Figure 3:**
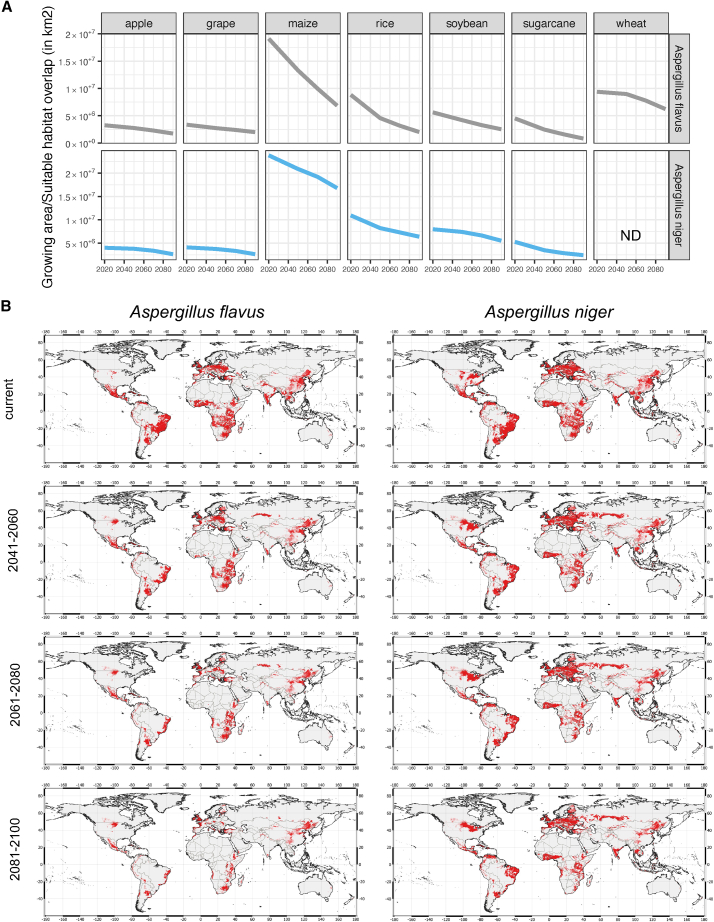
Suitable habitat will have lower overlap with crop-growing regions (A) Quantification of the km^2^ overlap between crop growing regions from CROPGRIDS and the suitable habitat for *A. niger* and *A. flavus*. ND is not done as *Aspergillus niger* has not been reported to cause wheat infection. (B) Map overviews of *A. flavus* and *A. niger* across three different time horizons for the SSP585 model. Red is the overlap between habitat suitability and the crop growing region for maize.

Both *A. flavus* and *A. niger* are the causative agents of plant infections of many different crops. Using our MaxEnt model and land usage from CROPGRIDS,^57^ we established the habitat suitability of these two plant pathogens across 7 different crops: apple, grape, maize, rice, soybean, sugarcane, and wheat for the severe climate model (SSP585) (Figure 3A). Across all crops, a reduction in habitat suitability across the growing areas was observed. Most interestingly, a steep decline was observed for *A. flavus* on maize habitat and rice. The maize growth area and habitat overlap was estimated to be 19.1 million km^2^ currently, but would reduce to 13.3 million km^2^ in 2050, 9.9 million km^2^ in 2070, and 6.8 million km^2^ in 2090. This steep decline was not observed for *A. niger* of which the growth area and habitat overlap was estimated at 23.8 million km^2^ currently, to 20.9 million km^2^ in 2050, 19.1 million km^2^ in 2070, and 16.8 million km^2^ in 2090. For rice crops a similar trend was observed in which the *A. flavus* habitat was estimated at 8.8 million km^2^ currently, but would reduce to 4.8 million km^2^ in 2050, 3.2 million km^2^ in 2070 and 2.0 million km^2^ in 2090, while for *A. niger* it was estimated at 10.9 million km^2^ currently, to 8.2 million km^2^ in 2050, 7.3 million km^2^ in 2070 and 6.4 million km^2^ in 2090.

A detailed spatial overview of these overlaps was generated, which revealed that for maize growing areas and *A. flavus* habitat, the main regions that showed a reduced overlap were located across South America and Africa (Figure 3B). However, habitat suitability in the Northern Hemisphere was mostly retained. A similar trend was observed for *A. niger,* but with a smaller effect. Some maize growing regions in Africa and South America would not be considered suitable, but habitats across the Northern Hemisphere, including India and Mexico was maintained. *A. flavus* habitat within rice growing regions was severely reduced and would in 2100 only be maintained in China and small regions in Africa (Figure S8). However, for *A. niger,* larger regions across South America (Brazil) and West Africa would be retained by 2100.

**Figure 4:**
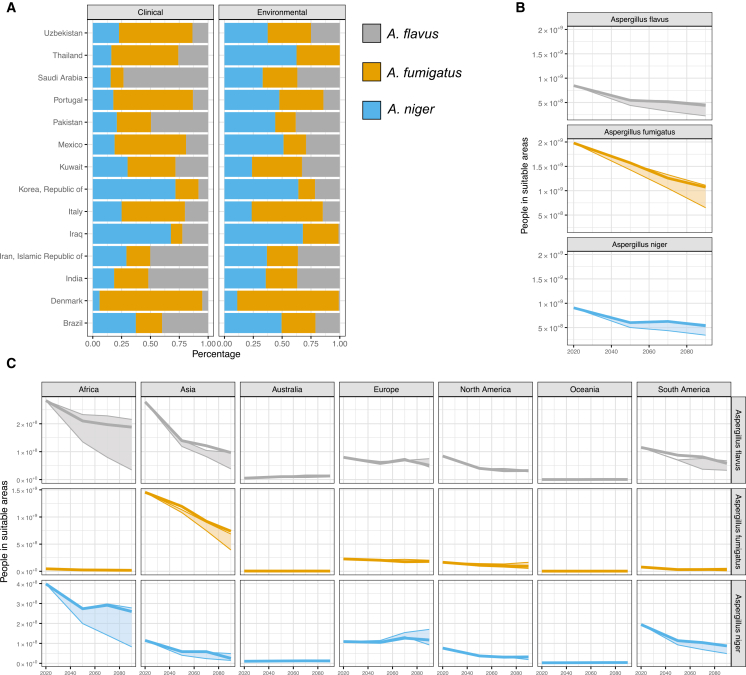
The epidemiological landscape of invasive aspergillosis is predicted to shift due to climate change (A) Correlation of the relative frequency of *Aspergillus* species found in the literature, where one report of clinical frequencies and one of the environmental frequencies could be found. Blue is *A. niger*, Orange *A. fumigatus,* and gray *A. flavus*. Shaded bands are the 95% confidence intervals. (B) People living in a suitable habitat for the three *Aspergillus* species until the 2100 time horizon. The solid line is the moderate scenario SSP245, while the bands represent the SSP585 and SSP126 models. (C) People living in suitable habitats are broken down into continents for the three *Aspergillus* species until the 2100 time horizon. Australia was considered separate in this analysis. The solid line is the moderate scenario SSP245, while the bands represent the SSP585 and SSP126 models.

Next, we wanted to know if a change in habitat could result in a change in causative agents of aspergillosis in the clinic. To assess the link between environment and clinical distribution of *Aspergillus* species, we found literature where at least one report contained relative prevalence in invasive aspergillosis of *A. niger, A. flavus,* and *A. fumigatus,* and at least one report from these Aspergilli and their relative prevalence in soils (Figure 4A). This resulted in 14 countries in which we could find literature with these data. This showed that species distribution from clinical samples (invasive pulmonary aspergillosis) generally correlated with the species distribution (*A. flavus* r(12) = 0.74, *p* = 0.002, *A. fumigatus* r(12) = 0.66, *p* = 0.011, *A. niger* r(12) = 0.40, *p* = 0.058) in the environment.

Given that habitat suitability and causative agents of invasive aspergillosis are correlated, we sought to model how many more people will be living in suitable areas for these *Aspergillus* species. We combined our MaxEnt model with a 1 km spatial model of population density across the same climate models (SSP126, SSP245, and SSP585).^58^ Currently, 846 million, 1.98 billion, and 905 million people live in suitable habitats for *A. flavus*, *A. fumigatus,* and *A. niger*, respectively (Figure 4B). Generally, fewer people will live in a suitable habitat for all three fungi. The largest effect is in suitable habitat for *A. fumigatus,* as this will be reduced to 650 million (SSP585 2081–2100) – 1.1 billion (SSP126 2081–2100), a reduction of 45–75%. The smallest effect will be on the number of people living in suitable areas for *A. niger*. Under the least severe climate model (SSP126), this will reduce to 562 million by 2100 (38% reduction), while under the most severe climate model (SSP585), 345 million people will live in suitable areas for *A. niger* by 2100 (a 62% reduction).

However, a more detailed analysis of people living in suitable areas across different continents shows other patterns of potential exposure to these *Aspergillus* species (Figure 4C). The largest reduction of people living in suitable areas for all three *Aspergillus* species is in Africa, Asia, and South America. In Asia a steep reduction of people living in suitable habitat is noticeable; for *A. flavus* from 278 million to 38 million (SSP585) – 98 million (SSP126), *A. fumigatus* from 1.5 billion to 392 million (SSP585) – 686 million (SSP126), and *A. niger* from 115 million to 14.5 million (SSP585) to 49 million (SSP126). In Africa lower numbers of people are already living in suitable areas for *A. fumigatus* (45 million) compared to *A. flavus* (283 million) and *A. niger* (398 million), but a reduction in people living in suitable habitats for *A. flavus* (34–216 million) and *A. niger* (83–278 million) is predicted, especially in the more severe SSP585 model. In Europe, a consistent number of people living in *A. flavus* (80 million currently versus 75 million) and an increase in people living in *A. niger* (109 million currently versus 170 million), suitable habitat will only be seen in the SSP585 model. Interestingly, an increase in people living in *A. flavus* suitable habitat across Australia is observed across all three climate models; 5 million currently, 10.2 million in SSP126, 12.8 million in SSP245, and 16.2 million people in the SSP585 model (Figure 4C).

In summary, we have generated a MaxEnt model for three *Aspergillus* species that are of relevance in plant infections and infections of humans and animals. We have shown that this model correlates with experimental culture-based data available and that our model can be used to predict potential future outcomes along different climate scenarios. This model showed that all three *Aspergillus* species will move more polewards and become more prevalent in the Northern hemisphere, while the less suitable habitat will be presented across the Southern Hemisphere. We show this can potentially impact plant infections and human infections and provide data that can be used to inform future surveillance strategies.

## Discussion

In this study, we have used a MaxEnt modeling approach to assess how the geographical distributions of three *Aspergillus* species; *A. fumigatus*, *A. flavus*, and *A. niger*, are likely to shift in response to climate change. This MaxEnt model, supported by global metabarcoding data and climate variables, highlight trends in current and future environmental suitability for these species. Notably, *A. flavus* and *A. niger* are more prevalent in tropical and subtropical climates with higher mean temperatures, whereas *A. fumigatus* shows greater suitability in cooler, temperate regions, as has been previously reported in the literature.^12^^,^^27^^,^^30^^,^^59^ *A. fumigatus* has been previously found in low concentrations in soils in New Zealand, a temperature zone in the southern hemisphere, and soils in Iceland, highlighting its potential to establish more northward and further expand.^60^^,^^61^ Our literature review supports a positive correlation between environmental suitability and clinical prevalence of *Aspergillus* species, suggesting that shifts in habitat suitability may result in changing patterns of aspergillosis worldwide.^62^^,^^63^ This is particularly concerning given the role of *A. flavus* and *A. niger* in both invasive human infections and crop contamination, especially as their environmental niches expand or shift.

While this study focuses on MaxEnt species distribution modeling, we acknowledge that alternative modeling approaches are available, including generalized linear models (GLM),^64^ gradient boosted models (GBMs).^65^^,^^66^ and random forest (FR) machine learning approaches.^67^^,^^68^^,^^69^ However, MaxEnt offers several advantages,^70^ including its ability to handle presence-only data effectively^71^; it is robust with relatively small sample sizes; incorporates regularization techniques to avoid model overfitting, and produces transparent and interpretable outputs.^72^ It is also widely used and well validated, having been effectively tested across taxa and geographies.^73^^,^^74^^,^^75^^,^^76^^,^^77^ In addition, here we have used one climate model, the HadGEM3-GC31-LL model.^78^ Over 40 different climate models are currently available with slightly different outcomes across the tested timelines.^79^ The HadGEM3-GC31-LL has shown a high climate sensitivity in the CMIP6 models, which has been debated as to whether these are inconsistent with evidence from historical records.^80^^,^^81^ Further research using other modeling approaches is required to come to a better understanding of the sensitivity of our analysis.

The MaxEnt modeling approach offers great potential in habitat suitability assessment; several methodological limitations are acknowledged. Firstly, highly customized MaxEnt models may become overly complex, leading to potential overfitting thus resulting in weakened predictive accuracy and ability to extrapolate to undersampled areas or new time horizons.^82^^,^^83^ Moreover, MaxEnt also assumes that the presence data used in the model are geographically representative of the true species distribution. However, occurrence records typically exhibit spatial bias due to uneven sampling and/or reporting efforts, which we highlighted as no samples were available, for example, the Amazon, northern Russia, Alaska, and the Sahara dessert.^84^^,^^85^^,^^86^ Finally, MaxEnt uses a presence-only modeling framework, therefore generating relative, rather than absolute, suitability. Therefore, consideration and careful interpretation are required when comparing multiple species or across environments. MaxEnt and other modeling approaches do not account for biotic interactions, microenvironmental variability, or genetic adaptation. For example, we do not account for the potential evolution of thermotolerance, virulence, or fungicide resistance, which could drastically alter species distributions or the ability to cause infections.^87^^,^^88^^,^^89^

In addition, although we focused on climate variables, other abiotic factors, such as soil composition, pH, and anthropogenic land use, undoubtedly influence *Aspergillus* ecology.^90^ *A. fumigatus*, in particular, is strongly associated with thermogenic environments rich in decaying organic matter, such as compost heaps, where temperatures can exceed 50°C during active decomposition.^91^^,^^92^ Surveys across the UK found elevated levels of *A. fumigatus* across compost bags, heaps, and garden plots treated with compost, which was associated with antifungal-resistant isolates.^54^^,^^93^ These conditions provide a unique niche for *A. fumigatus*, enabling high sporulation and aerial dispersal, especially when compost is disturbed.^92^^,^^94^ In contrast, *A. flavus* and *A. niger* are more frequently isolated from multiple types of soils, with high organic content, lower nitrogen levels, and acidic to neutral pH.^95^^,^^96^ Soil pH has been shown to influence fungal community structure, with *A. niger* thriving in acidic conditions.^97^^,^^98^ Another layer of uncertainty stems from population projections in suitable habitats. While we estimate increasing exposure risk in some regions, these are modeled on current species-environment relationships and may not capture future human behavior, people at risk of developing fungal infections, or agricultural changes that would render plants at risk of infection.

In addition to long-term climatic changes, seasonal variation and extreme weather events are likely to play an important role in shaping the distribution of *Aspergillus* species.^99^ Seasonal dynamics influence growth and spore release, particularly through cycles of rainfall and temperature shifts, as is seen with other fungal pathogens.^100^^,^^101^^,^^102^ The MaxEnt model identified precipitation seasonality as a key predictor of habitat suitability, particularly for *A. flavus* and *A. niger*, suggesting these species are more present in areas with increased wet-dry cycles. Furthermore, extreme weather events such as droughts, floods, and heatwaves, which are expected to increase in frequency and intensity, can contribute to higher levels of fungal spores within the air.^92^^,^^103^ Past studies have observed spikes in aspergillosis cases following natural disasters.^104^^,^^105^

While our MaxEnt model provides a prediction of suitable habitat overlap within crop-growing regions, it does not account for climate change directly impacting the crop-growing regions. Several modeling attempts have shown that regions will become unsuitable to grow rice,^106^^,^^107^^,^^108^ wheat,^109^^,^^110^ and maize^111^^,^^112^ under different climate scenarios. In addition, the differential virulence of species and the occurrence of *Aspergillus* species across different crops and their disease has not been accounted for. While this would ideally be done, current epidemiological data from across the world remain sparse. Future work combining crop models, virulence data, and epidemiology would provide a more detailed approach to model plant infections in a changing world.

Historically, invasive aspergillosis was primarily a concern for immunocompromised individuals, such as transplant recipients or those undergoing chemotherapy.^113^ Our MaxEnt model does not take into account the changing patient population or emerging novel risk factors for aspergillosis. Examples of recently associated risk factors include COVID-19 and severe influenza, leading to COVID-19-associated pulmonary aspergillosis (CAPA) and influenza-associated pulmonary aspergillosis (IAPA), collectively termed viral-associated pulmonary aspergillosis (VAPA).^114^^,^^115^ These diseases have been increasingly recognized in intensive care settings, where patients often experience prolonged ventilation and receive corticosteroids or other immunomodulatory treatments.^116^

Despite these caveats, this work represents a valuable step in modeling the climate-driven shifts in *Aspergillus* ecology. By combining environmental metagenomic sequencing and modeling with clinical and environmental prevalence data, we highlight the importance of proactive monitoring in a changing world. The expanding and shifting range of these fungal pathogens, exacerbated by climate change, reinforces the urgency of a One Health approach to infectious disease surveillance.

### Limitations of the study

This study has several limitations that should be considered when interpreting the findings. First, species occurrence data were derived primarily from global metabarcoding datasets, which rely on ITS sequencing that does not reliably resolve *Aspergillus* species beyond the section level, introducing uncertainty in species-level attribution, particularly within sections Nigri and Flavi. In addition, the presence-only nature of MaxEnt modeling produces relative suitability rather than true probability of occurrence and is sensitive to spatial sampling bias, which is evident in the underrepresentation of large regions such as the Amazon basin, Sahara, northern Russia, and the Arctic regions. Also, biotic interactions, microclimatic conditions, land management practices, and point-source habitats such as composting sites are not explicitly modeled, despite their known importance for *Aspergillus* ecology. Future projections rely on a single global climate model and do not capture inter-model variability present across CMIP6 ensembles, which can influence regional predictions. Finally, while the spatial modeling framework provides quantitative projections of potential future habitat suitability, it remains a theoretical representation of complex ecological systems, and the actual real-world impact on *Aspergillus* exposure, disease burden, and crop losses will ultimately depend on future environmental, biological, agricultural, and societal factors that require surveillance and experimental validation.

## Resource availability

### Lead contact

Requests for further information and resources should be directed to and will be fulfilled by the lead contact, Norman van Rhijn (norman.vanrhijn@manchester.ac.uk).

### Materials availability

This study did not generate new unique reagents.

### Data and code availability

• This article analyses existing, publicly available data, accessible at https://globalfungi.com. Other databases used have been mentioned in the relevant section in the STAR Methods.
• This article does not report original code.
• Any additional information required to reanalyze the data reported in this article is available from the lead contact upon request.

## Acknowledgments

We thank M. Bromley and M. Brockhurst (10.13039/501100000770UoM) for their insightful feedback on this work. This work was supported by the 10.13039/100010269Wellcome Trust (grant: 226408/Z/22/Z).

## Author contributions

C.U. conceptualization, formal analysis, investigation, methodology, and writing – original draft. J.S. formal analysis, investigation, methodology, and writing – original draft N.v.R conceptualization, formal analysis, funding acquisition, supervision, and writing – original draft.

## Declaration of interests

The authors declare no competing interests.

## Declaration of generative AI and AI-assisted technologies in the writing process

During the preparation of this work, the authors used Google Gemini and NotebookLLM in order to correct typos and grammar errors and generate the basis of the graphical abstract, respectively. After using this tool, the authors reviewed and edited the content and take full responsibility for the content of the publication.

## STAR★Methods

### Key resources table

**Table:** 

REAGENT or RESOURCE	SOURCE	IDENTIFIER
**Deposited data**		

GlobalFungi v5	Vetrovsky et al. 2020	globalfungi.com
WorldClim 2	Fick and Hijmans 2017	worldclim.org
HadGEM3-GC31-LL	O'Neill et al. 2016	https://www.wdc-climate.de/ui/cmip6?input=CMIP6.CMIP.MOHC.HadGEM3-GC31-LL
CROPGRIDS	Tang et al. 2024	https://figshare.com/articles/dataset/CROPGRIDS/22491997
Population distributions under SSP models	Wang et al. 2022	https://figshare.com/articles/dataset/Projecting_1_km-grid_population_distributions_from_2020_to_2100_globally_under_shared_socioeconomic_pathways/19608594/2

**Software and algorithms**		

SPSS Statistics 24	IBM	–
ArcGIS Pro v3.4.2	Esri	–
MaxEnt v3.4.4	https://biodiversityinformatics.amnh.org/open_source/maxent/	–
RStudio v2024.09.0 + 375	Posit PBC	–

### Method details

#### Data acquisition

To gather metabarcoding sequencing data on *Aspergillus* species, the GlobalFungi database (release 5.0) was used.^46^ Search by taxonomy on *Aspergillus fumigatus*, *Aspergillus flavus* and *Aspergillus niger* was used. Raw data containing sample ID, latitude and longitude, sample type and ITS total were exported following data quality control. Data from aquatic and air samples were removed as well as manipulated samples. To remove potential datapoints that resulted from low level contamination only datapoints with over 10 sequencing reads attributed to each species were maintained. Data were stored and analysed using IBM SPSS Statistics 24.

Current and future bioclimate variables were obtained from the WorldClim data archive.^117^ Initially a total of 19 bioclimate variables were downloaded with a spatial resolution of 5 arc-min (10km^2^) were selected for analysis. Initially, a baseline MaxEnt model was constructed with all 19 variables to assess contribution percentage, and Pearson correlation coefficients between variables were calculated. Variables demonstrating a correlation exceeding ± 0.8 were investigated and the variable with the lower contribution in the baseline model was excluded. Ultimately, seven WorldClim bioclimate variables - Annual Mean Temperature (bio_01), Mean Diurnal Range (bio_02), Temperature Annual Range (bio_07), Annual Precipitation (bio_12), Precipitation of Driest Month (bio_14), Precipitation Seasonality (bio_15) (which is calculated as the coefficient of variation of monthly precipitation) and Precipitation of Coldest Month (bio_19) were retained for MaxEnt modelling.

The future climate data used in this study comes from the Sixth iteration of the Coupled Model Intercomparison Project.^118^ Specifically, we used the HadGEM3-GC31-LL future climate dataset for 3 shared socioeconomic pathways (SSPs; SSP 126, SSP 245 and SSP 585) for 3 future time horizons: 2014-2060, 2061-2080 and 2081-2100.

Data on future human population density was obtained from projections at a 30 arc-seconds (1km) spatial resolution until 2100 under different SSP models.^58^ Data on spatial distribution of growing different crops (5.6km resolution) was obtained from CROPGRIDS.^57^ A selection of crops to focus on was chosen at the top 10 highest value crops globally. Data intersections and maps were generated using ArcGIS Pro v3.4.2.

#### Literature review

A literature search was performed using several search terms for each individual country from the WHO country list; “country name” AND aspergillosis OR aspergillus, as well as “country name” AND aspergillus AND soil. Articles were manually curated and included when all three species were identified in the data, which allowed comparison of species prevalence. Articles referring to specific substrates (food items, fruits etc) were excluded and only data on soil species distributions were included for environmental prevalence of *Aspergillus* species. For clinical prevalence only data from invasive aspergillosis was used to make articles comparable.

### Quantification and statistical analysis

#### Model generation

All pre-processing was undertaken in ArcGIS Pro 3.2. Occurrence data were cleaned, projected to a uniform coordinate system, and spatially thinned to reduce autocorrelation. Environmental predictor variables were reprojected, resampled, and clipped to a consistent spatial resolution and study extent.

Using MaxEnt v3.4.4, separate species-specific models were generated using GPS coordinates for *Aspergillus fumigatus*, *Aspergillus flavus* and *Aspergillus niger* individually. For each individual species model, 80% of occurrence records were used for model training and 20% for independent validation. Model complexity was explicitly defined by testing feature class (FC) combinations of L, H, LQ, LQH, and LQHPT with regularization multipliers (RM) of between 0.5 and 4 at 0.5 intervals. The maximum number of iterations was set to 500, and a convergence threshold of 0.00001 was applied to ensure model stability while minimising overfitting.

Each model used 10,000 background (pseudo-absence) points, spatially constrained by species-specific bias files to match the sampling structure of occurrence data. Bootstrap replication (n = 10) was employed, and outputs were generated in Cloglog format to facilitate interpretation of habitat suitability as relative probability of presence. For each species, input variable importance was assessed via the Jackknife test, and response curves were examined to evaluate ecological relationships. Jackknife test of regularised training gain was assessed to quantify the importance of each environmental variable in isolation as well as when it is removed from the dataset. Model performance was quantified using the area under the receiver operating characteristic curve (AUC).

Following MaxEnt calibration, resulting suitability layers were imported into the ArcGIS Pro for further spatial analysis. Continuous Cloglog outputs were reclassified using the maximum training sensitivity plus specificity (MTSPS) threshold to delineate suitable habitat areas. In ArcGIS, the Reclassification Tool was used to divide habitats into non-suitable (0-MTSPS value) and suitable (MTSPS value-1). The future suitable habitats were generated by overlaying habitats using the “Intersect” function.

Maps and additional spatial analysis were executed using ArcGIS Pro v3.4.2. All other data was visualised using Rstudio (v 2024.09.0+375) and ggplot2.

Differences between bioclimatic variables were assessed via One-way ANOVA with post-hoc Tukey’s Honest Significant Difference. P<0.05 was considered significant.
