# Supplementary material for: Climate change-driven geographical shifts in Aspergillus species and the implications for plant and human health
Source: iScience. 2026 Feb 5;29(3):114911. doi: 10.1016/j.isci.2026.114911 (PMC12933601; doi:10.1016/j.isci.2026.114911)
Supplement: Document S1. Figures S1–S8, and Table S1 [file mmc1.pdf]

iScience, Volume *29*

Supplemental information

Climate change-driven geographical shifts in *Aspergillus* species and the implications for plant and human health

Christopher Uzzell, Jennifer Shelton, and Norman van Rhijn

Fig S1 Global distribution of datapoints used for the MaxENT model, Related to Figure 1. A *A. flavus* occurrences and locations (n=871) **B** *A. fumigatus* occurrences and locations (n=319) **C** *A. niger* occurrences and locations (n=1021).

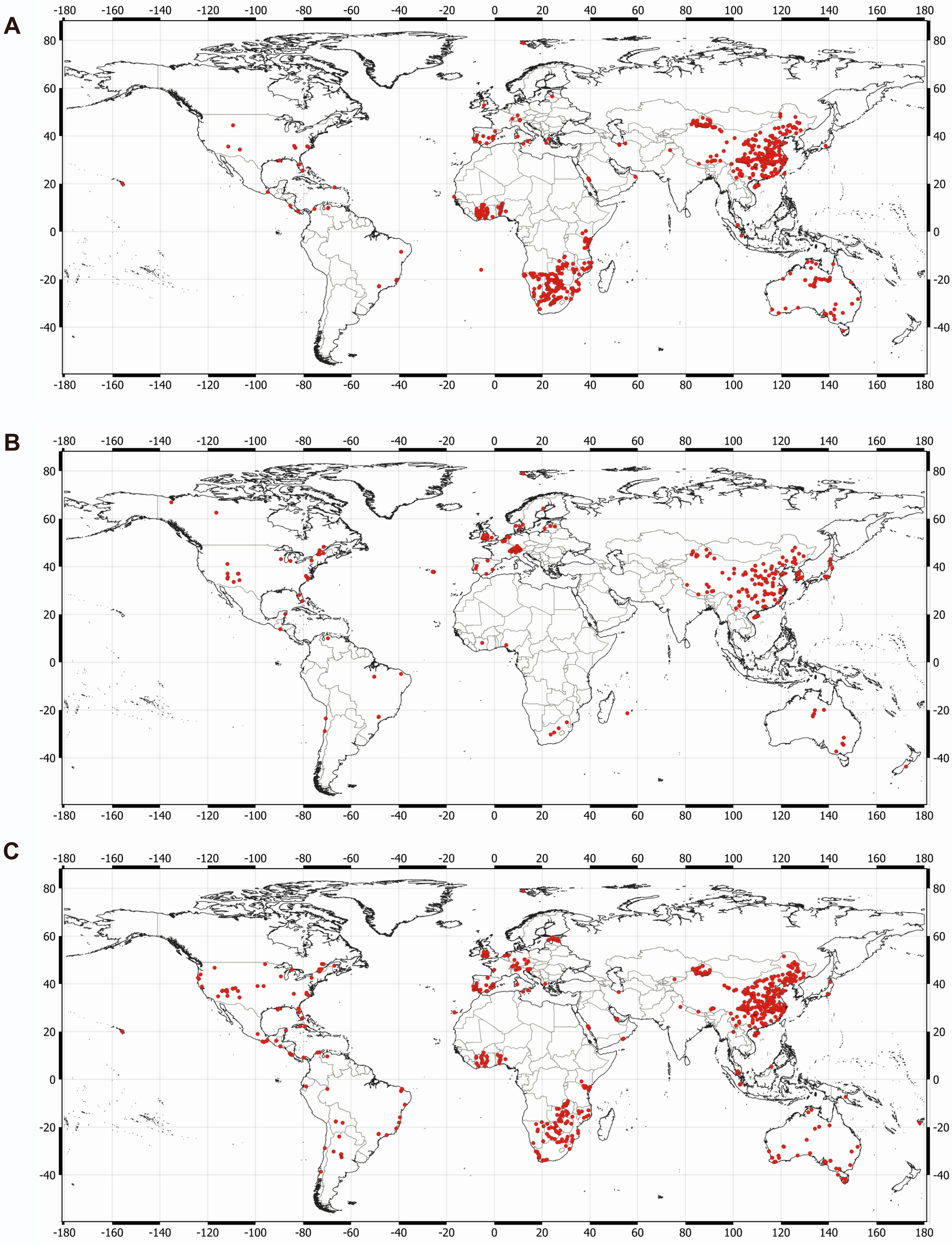

Fig S2 Correlation matrix for bioclimate variables, Related to Figure 1. A Pearson correlation matrix for tall bioclimate environmental variables and correlation to each other. Red is negative correlation, blue is positive correlation. Red boxes are the bioclimate variable that are retained. **B** Highlighted bioclimate variables that are retained. Blue is a positive correlation, red is negative.

A

B

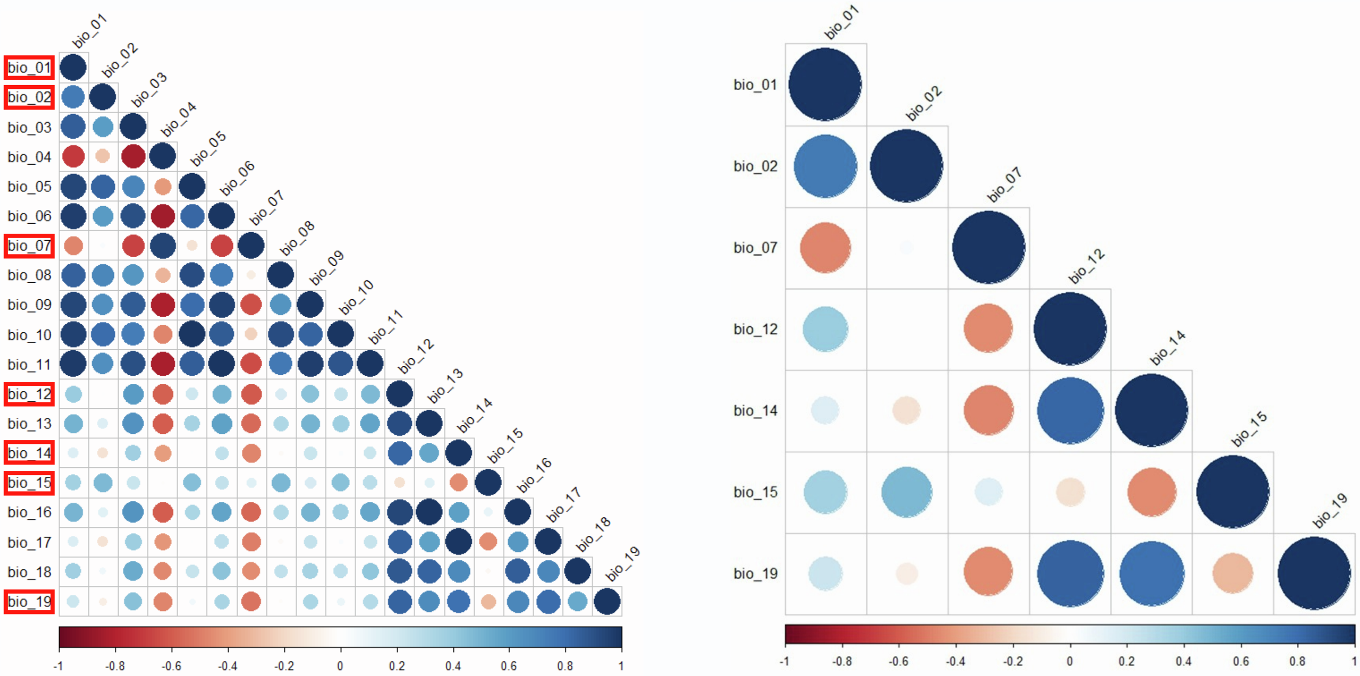

Fig S3 Model evaluation, Related to Figure 1. MaxEnt AUC ROC curves for (a) *A. flavus,* (b) *A. fumigatus* and (c) *A. niger*. Red line and grey area show the mean and 1STDof the 10 replicate model runs, respectively. Light blue represents the cumulative area under the curve (AUC) with test statistics shown. Jackknife tests for variable importance of (d) *A. flavus,* (e) *A. fumigatus* and (f) *A. niger* habitat suitability distribution.

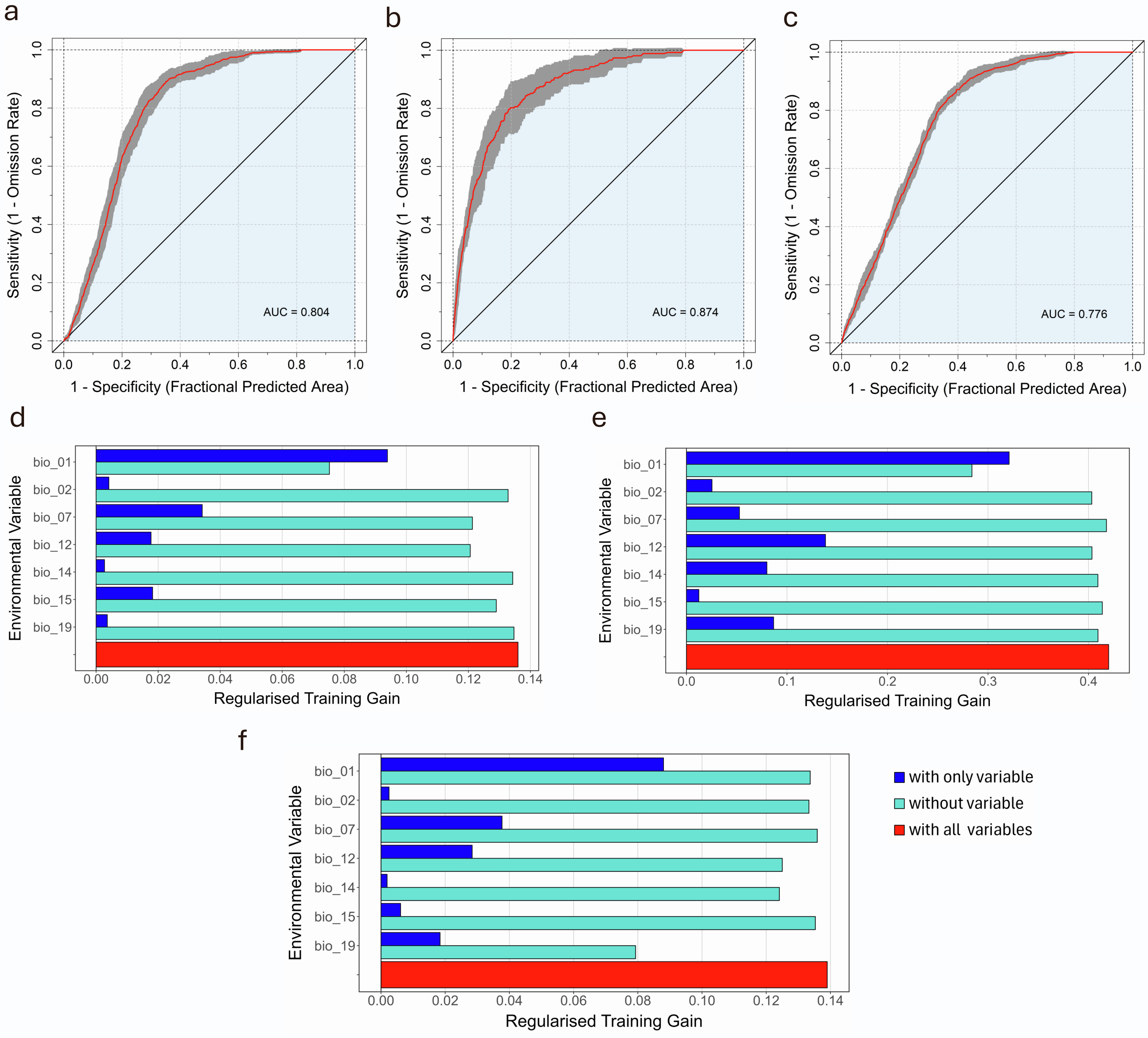

Fig S4 Kernal density plots of bioclimate variables, Related to Figure 1. Kernel density plots of *A. flavus, A. fumigatus* and *A. niger* for their occurrences associated with each bioclimate variable. Colours indicate each species and the height of each peak represent the relative frequence at which these species occurred.

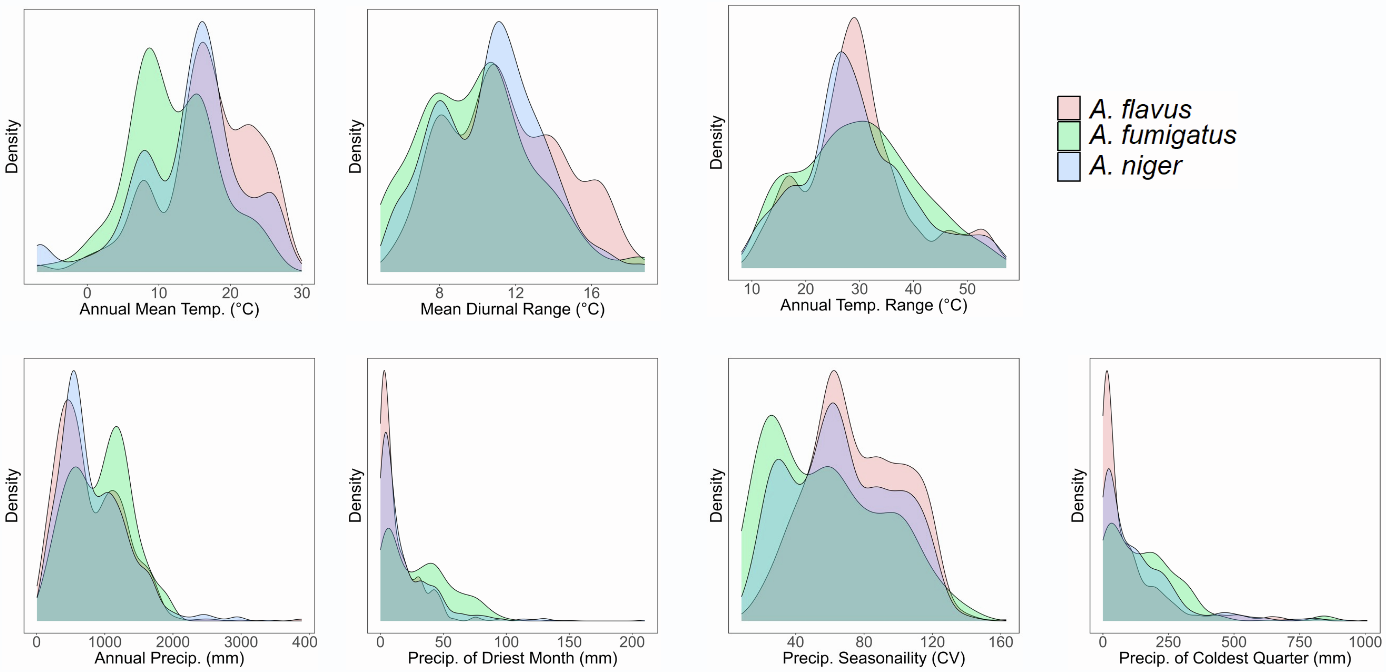

Fig S5 Ridgeplots of value distribution of each species in SoilGrid layers, Related to Figure 1. Each soil parameter from SoilGrids was assessed for the occurrence of the three species. Red colour is *A. flavus*, green is *A. fumigatus* and blue equals *A. niger*. Lines show the mean of each plot for each species.

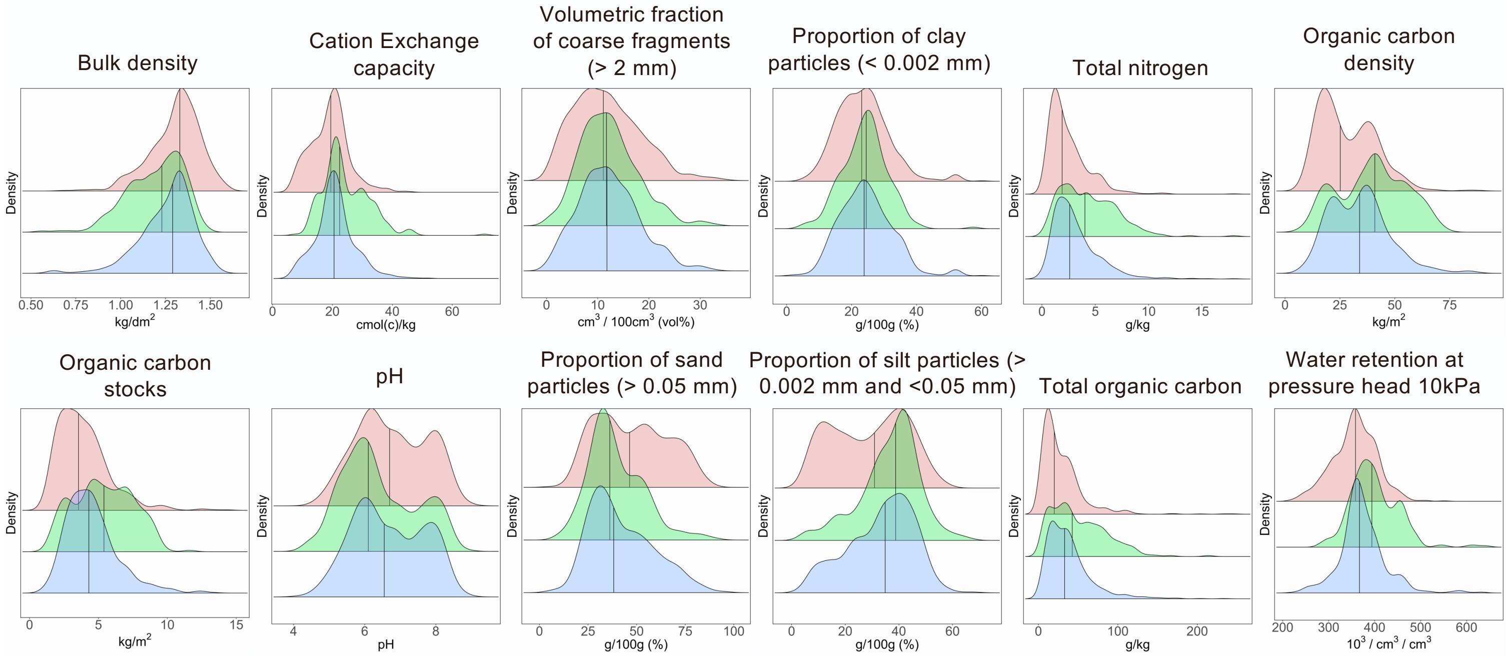

Fig S6 Maps of all climate models analysed across the three timelines, Related to Figure 2. Maps of habitat suitability across the world for three Aspergillus species. Red is considered suitable and grey unsuitable.

Aspergillus flavus

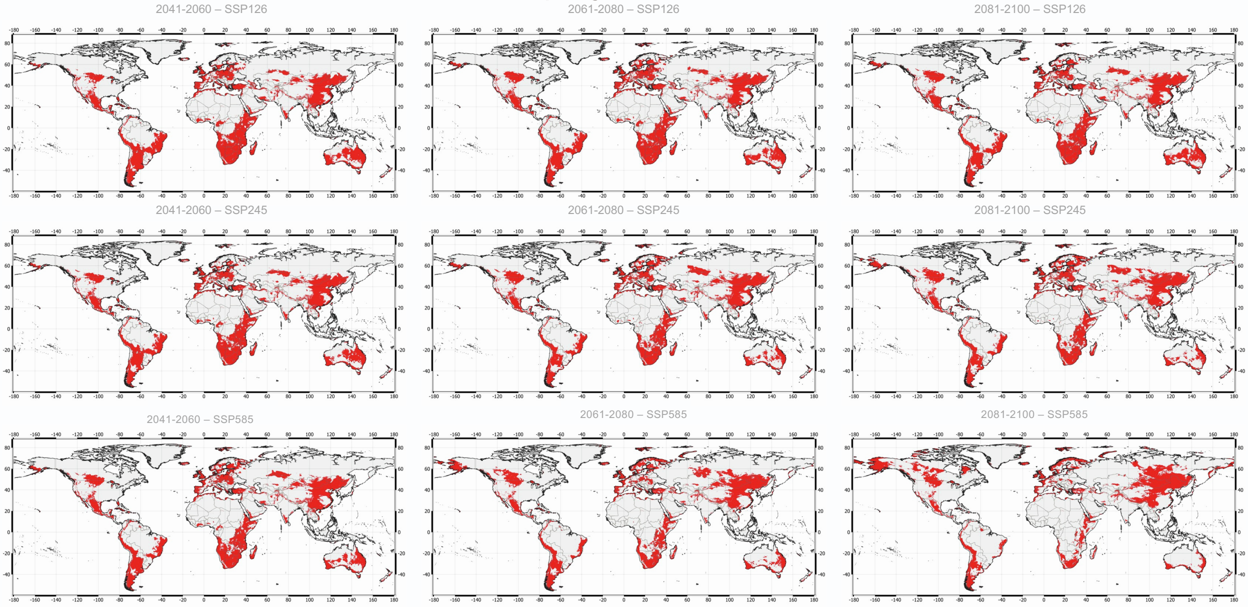

Aspergillus fumigatus

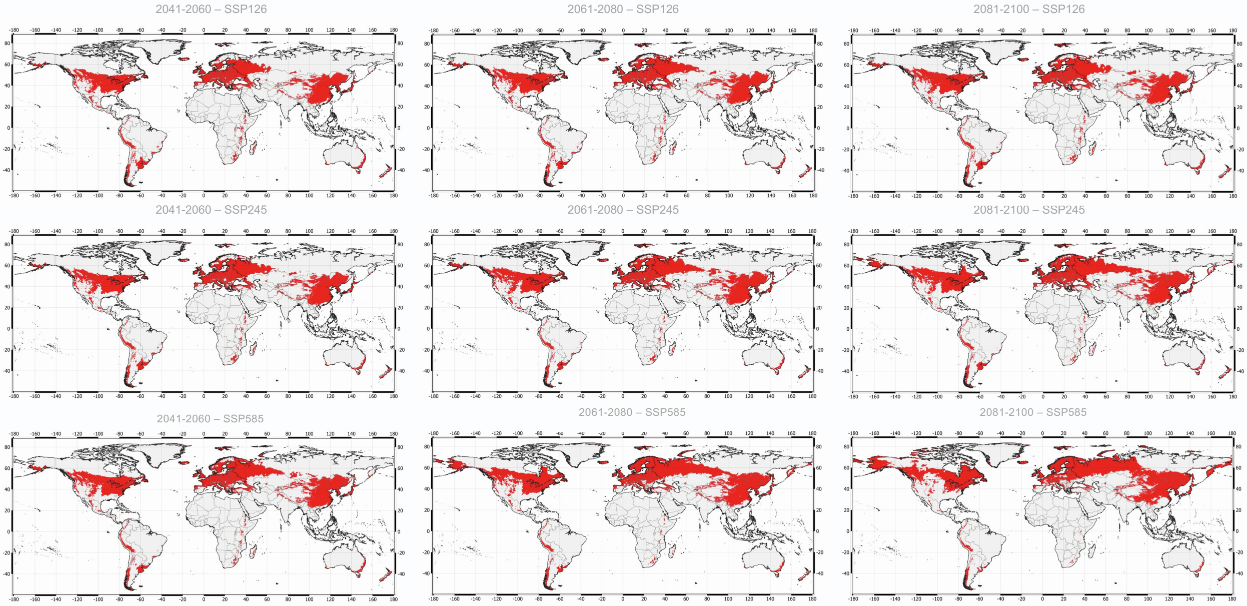

Aspergillus niger

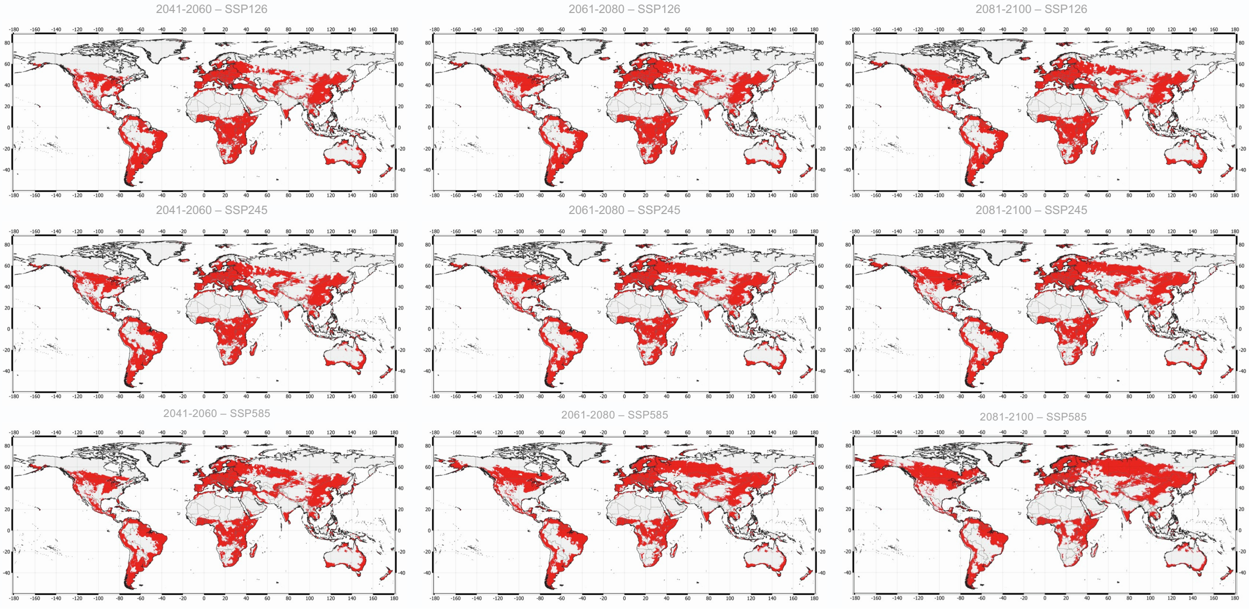

Fig S7 Suitability plotted across latitude, Related to Figure 2. Suitability from the MaxENT model mapped across latitude for the three *Aspergillus* species across the three different climate models across different time horizons.

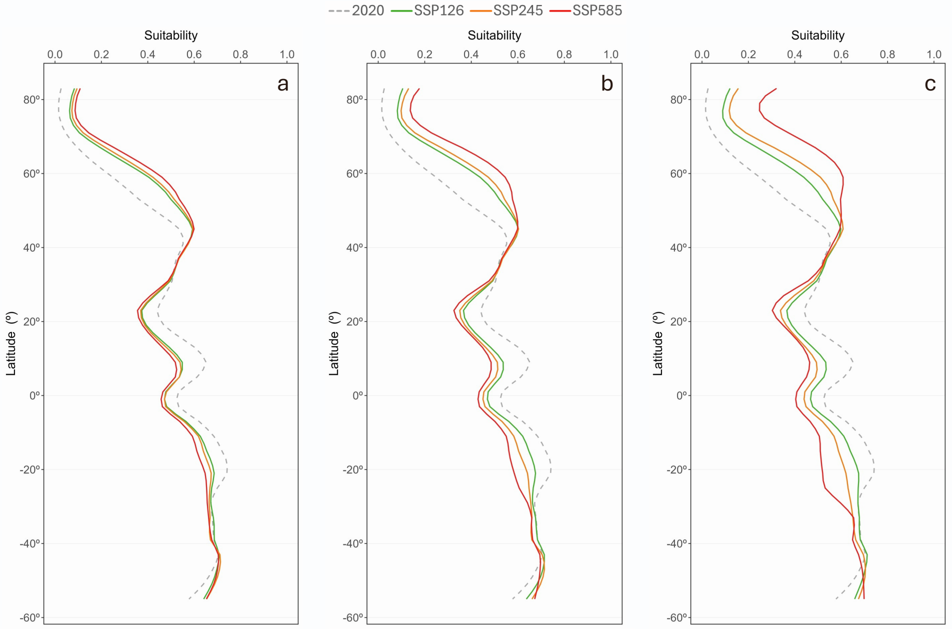

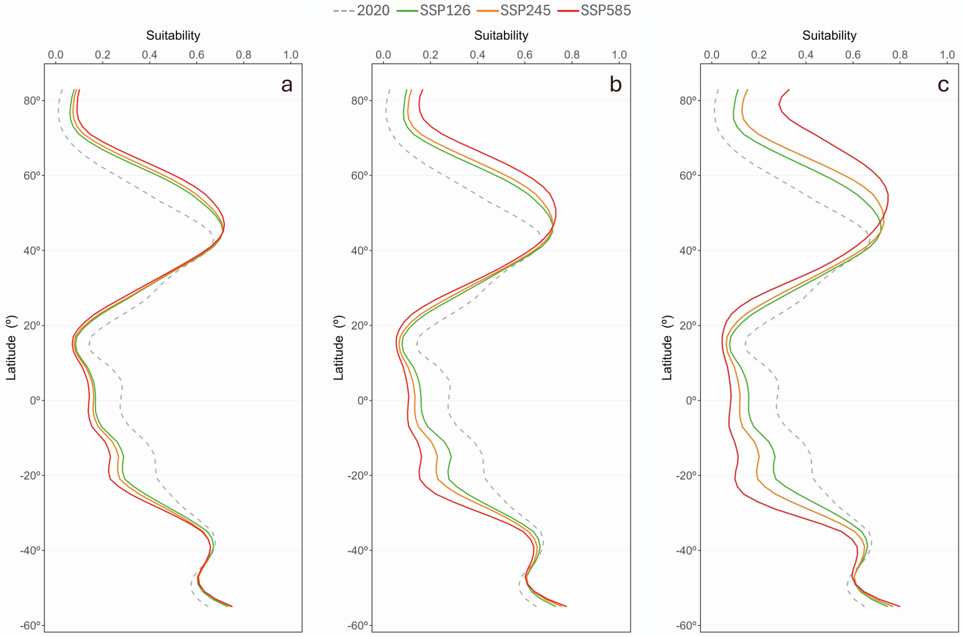

Fig. #. Mean logistic output (probability of presence) according to latitude for *A. fumigatus* under current and future climate modelling scenarios for various SSPs. Future time horizons: (a) 2041-2060, (b) 2061-2080, (c) 2081-2100.

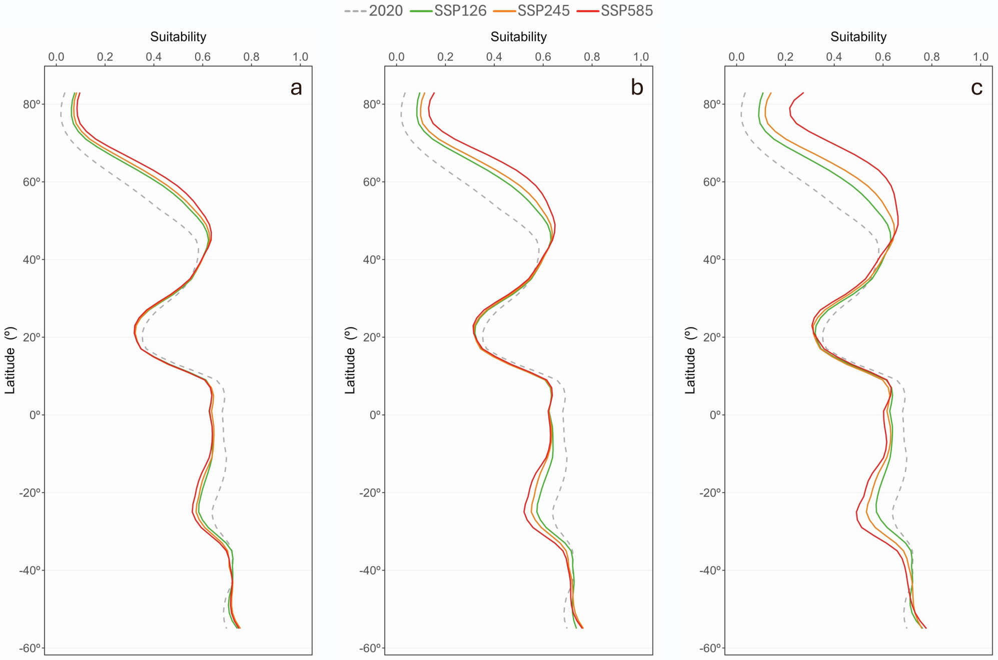

Figure S8 Habitat overlap with rice growing regions, Related to Figure 3. Habitat suitability and overlap with rice growing regions is indicated in red for *A. flavus* and *A. niger.*

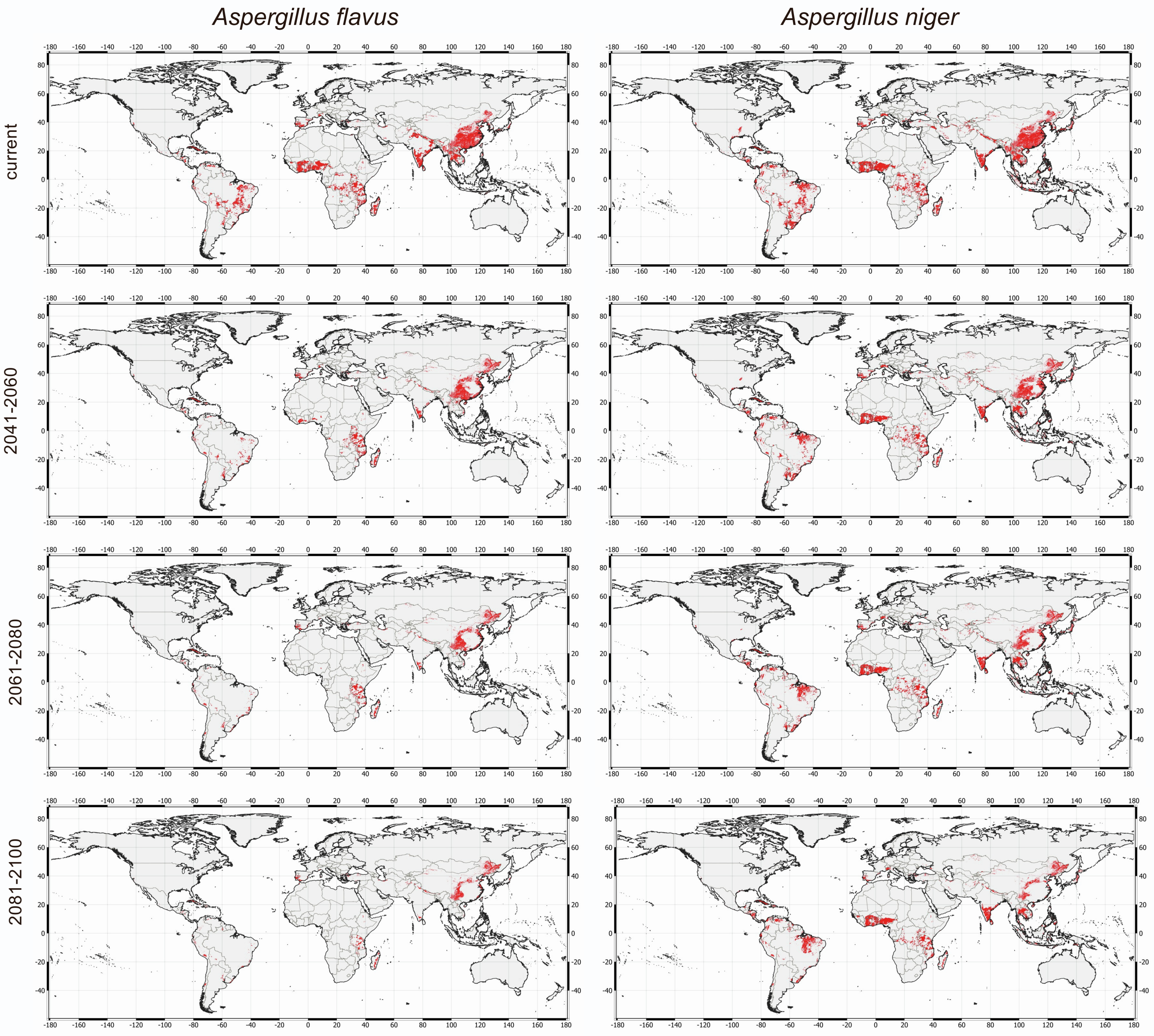

Table S1 Population exposure for three modelled *Aspergillus* species under the three tested SSP models divided per continent, Related to Figure 4

	CONTINENT	2020	2041_2060_SSP126	2061_2080_SSP126	2081_2100_SSP126	2041_2060_SSP245	2061_2080_SSP245	2081_2100_SSP245	2041_2060_SSP585	2061_2080_SSP585	2081_2100_SSP585
Global Population	Africa	1,360,986,051	1,788,129,968	1,946,691,602	1,943,399,522	2,034,261,978	2,394,843,955	2,601,221,292	1,762,177,981	1,900,647,781	1,888,151,138
	Australia	21,131,163	34,920,864	39,913,489	40,730,892	34,383,933	39,243,027	41,068,677	42,202,893	53,732,707	61,242,955
	Europe	619,079,630	632,615,322	628,528,432	589,740,540	619,434,361	612,182,357	590,560,059	702,148,180	764,674,756	797,809,643
	North America	616,540,269	685,010,933	715,301,452	711,483,909	701,379,103	743,151,154	757,044,001	744,059,559	842,015,806	923,166,278
	Oceania	7,411,470	8,770,799	9,061,833	8,842,854	9,081,022	9,564,275	9,658,468	9,442,573	10,501,119	11,219,955
	South America	456,200,383	446,996,786	414,152,473	354,851,455	487,293,735	479,408,383	447,639,168	438,832,665	402,435,713	342,392,626
	Asia	4,836,558,042	4,874,950,621	4,494,727,984	3,826,077,228	5,284,976,655	5,178,866,185	4,807,247,137	4,869,986,199	4,491,345,877	3,833,821,647
	Total	7,917,907,006	8,471,395,294	8,248,377,266	7,475,126,400	9,170,810,788	9,457,259,337	9,254,438,801	8,568,850,051	8,465,353,759	7,857,804,242
Asp Flav HS pop	CONTINENT	2020	2041_2060_SSP126	2061_2080_SSP126	2081_2100_SSP126	2041_2060_SSP245	2061_2080_SSP245	2081_2100_SSP245	2041_2060_SSP585	2061_2080_SSP585	2081_2100_SSP585
	Africa	282,958,873	232,935,857	228,495,517	215,667,029	210,061,192	197,086,637	188,013,316	135,102,538	79,894,102	34,628,617
	Australia	5,073,427	10,811,119	13,209,646	10,209,280	10,445,877	10,645,234	12,850,372	11,212,976	16,345,250	16,254,500
	Europe	79,817,494	61,357,835	75,893,831	44,606,590	57,988,566	70,570,895	51,330,328	64,908,064	68,733,823	75,004,074
	North America	84,328,100	38,845,145	39,987,396	33,650,820	40,203,894	31,708,438	32,323,560	39,857,120	36,359,853	27,747,940
	Oceania	224,454	186,857	239,293	358,517	146,499	587,510	429,750	331,882	129,015	100,258
	South America	115,237,026	70,471,799	75,804,760	67,355,394	87,375,033	81,366,559	58,252,272	69,592,299	37,091,316	33,264,487
	Asia	278,505,032	141,054,766	104,221,664	97,974,732	139,387,356	121,205,559	96,684,333	118,713,300	82,164,275	38,178,431
	Total	846,144,407	555,663,378	537,852,106	469,822,362	545,608,417	513,170,832	439,883,931	439,718,180	320,717,633	225,178,308
		100	65.67004088	63.56504889	55.52508037	64.48171405	60.64813852	51.98686269	51.96727371	37.90341582	26.61227872
Asp Fum HS pop	CONTINENT	2020	2041_2060_SSP126	2061_2080_SSP126	2081_2100_SSP126	2041_2060_SSP245	2061_2080_SSP245	2081_2100_SSP245	2041_2060_SSP585	2061_2080_SSP585	2081_2100_SSP585
	Africa	45,415,589	27,186,064	24,684,628	20,525,198	24,401,811	21,809,223	17,584,148	15,208,796	3,565,549	1,330,954
	Australia	4,309,355	4,702,498	5,019,282	5,265,489	3,953,680	4,457,825	3,637,076	4,641,103	5,342,845	2,909,046
	Europe	227,458,878	212,406,466	219,732,920	203,580,741	203,541,941	175,382,553	180,805,821	203,896,352	193,792,108	189,764,160
	North America	165,509,085	141,676,990	134,515,423	164,771,393	112,520,902	100,295,542	92,508,786	93,452,767	77,210,578	52,583,985
	Oceania	2,288,467	2,281,071	2,574,775	2,719,880	2,346,017	2,598,044	2,563,668	2,879,476	2,644,557	2,716,592
	South America	77,851,362	36,865,290	40,226,075	28,329,191	31,094,727	31,943,281	37,615,944	26,934,698	19,026,818	8,529,674
	Asia	1,455,486,465	1,133,309,310	901,576,244	686,330,763	1,194,036,337	921,043,266	737,985,551	1,080,352,224	747,667,238	392,267,760
	Total	1,978,319,202	1,558,427,688	1,328,329,348	1,111,522,656	1,571,895,414	1,257,529,733	1,072,700,994	1,427,365,415	1,049,249,693	650,102,172
		100	78.77534052	67.14433882	56.18520282	79.4561066	63.56556271	54.222847	72.15040998	53.03743157	32.86133862
Asp Nig HS pop	CONTINENT	2020	2041_2060_SSP126	2061_2080_SSP126	2081_2100_SSP126	2041_2060_SSP245	2061_2080_SSP245	2081_2100_SSP245	2041_2060_SSP585	2061_2080_SSP585	2081_2100_SSP585
	Africa	398,167,002	277,366,403	297,687,057	277,643,253	273,723,463	291,358,744	259,574,308	199,153,018	141,359,255	82,632,176
	Australia	10,374,253	13,530,657	16,014,495	16,853,092	11,696,918	12,378,385	11,843,955	13,769,611	13,201,260	7,411,352
	Europe	109,156,716	109,959,331	133,721,453	92,325,503	104,931,500	126,257,669	117,023,644	114,499,986	154,303,777	170,155,963
	North America	76,282,905	38,303,709	36,024,844	35,807,484	37,098,957	30,838,881	32,198,598	37,762,546	32,389,347	18,716,725
	Oceania	2,606,154	2,964,416	3,085,920	3,400,119	3,023,021	3,625,838	3,777,297	3,485,637	3,474,750	2,404,970
	South America	194,478,898	116,985,894	100,628,958	87,630,102	113,464,095	104,031,714	87,424,992	92,346,807	69,049,076	48,707,739
	Asia	114,816,199	60,264,661	55,467,417	48,909,082	58,587,896	58,476,371	24,910,465	40,328,663	23,530,237	14,479,525
	Total	905,882,126	619,375,071	642,630,144	562,568,636	602,525,850	626,967,603	536,753,260	501,346,268	437,307,703	344,508,450
		100	68.3725899	70.93970899	62.1017481	66.51261049	69.21072676	59.25199809	55.34343306	48.27423905	38.0301631
